# Role of Natural Volatiles and Essential Oils in Extending Shelf Life and Controlling Postharvest Microorganisms of Small Fruits

**DOI:** 10.3390/microorganisms6040104

**Published:** 2018-10-05

**Authors:** Toktam Taghavi, Chyer Kim, Alireza Rahemi

**Affiliations:** Agricultural Research Station, Virginia State University, Petersburg, VA 23806, USA; ckim@vsu.edu (C.K.); a_rahemi@yahoo.com (A.R.)

**Keywords:** postharvest diseases, food borne pathogens, bacteria, fungi, food safety, plant extracts, small fruits, grape, strawberry, blueberry, raspberry, blackberry, essential oils

## Abstract

Small fruits are a multi-billion dollar industry in the US, and are economically important in many other countries. However, they are perishable and susceptible to physiological disorders and biological damage. Food safety and fruit quality are the major concerns of the food chain from farm to consumer, especially with increasing regulations in recent years. At present, the industry depends on pesticides and fungicides to control food spoilage organisms. However, due to consumer concerns and increasing demand for safer produce, efforts are being made to identify eco-friendly compounds that can extend the shelf life of small fruits. Most volatiles and essential oils produced by plants are safe for humans and the environment, and lots of research has been conducted to test the in vitro efficacy of single-compound volatiles or multi-compound essential oils on various microorganisms. However, there are not many reports on their in vivo (in storage) and In situ (in the field) applications. In this review, we discuss the efficacy, minimum inhibitory concentrations, and mechanisms of action of volatiles and essential oils that control microorganisms (bacteria and fungi) on small fruits such as strawberries, raspberries, blueberries, blackberries, and grapes under the three conditions.

## 1. Introduction

There are two major concerns for the handling and consumption of small fruits. First is their short shelf life, and second is food safety. Fresh produce in particular is gaining increasing attention in the current food safety and regulatory climate. Centers for Disease Control and Prevention [1], has developed a comprehensive list of food sources of all foodborne illnesses in the United States (U.S.) and fresh produce accounted for nearly half of illnesses among all types of foods reported during 1998–2008.

Standard small fruit production practices discourage growers from washing fruits after harvest and during storage. This may contribute to the buildup of food-borne bacteria and may pose a food safety risk to consumers since small fruits are eaten fresh. Also, small fruits have a very short shelf life due to their susceptibility to spoilage pathogens, physiological disorders and mechanical injury [2]. This is partly due to their high nutrient content and water activity, and low pH which make them susceptible to fungal attack. Thus, losses during harvest, handling and marketing are sometimes as high as 50% [3]. Pathogens may also produce mycotoxins that make the fruit harmful to consumers [4].

Low temperature storage is not adequate to extend fruit shelf-life for prolonged storage or for delivery to distant markets. Therefore, controlled temperature combined with high CO_2_ in modified atmospheres has been recommended to reduce spoilage [5]. Although small fruits can tolerate high CO_2_ levels in storage, it causes off-flavors in the long term [6], and application of fungicides is still the main method to control postharvest diseases in small fruit [6,7,8]. However, under optimal conditions for disease development, fungicides are not effective and growers may try to overcome this challenge by increasing fungicide applications, which in turn leads to higher residue levels in produce [7]. In the long term, the population of beneficial organisms declines as residual fungicides lead to the development of resistant fungi [6,8,9]. High levels of pesticide residues on berries are of particular concern because they are consumed fresh shortly after harvest. This has placed strawberries at the top of the “Dirty Dozen” list [10] and raised concerns about human health and pronounced oncogenic risk [8,11]. Other concerns with excessive pesticide application are increased cost, handling hazards, and threats to the environment [12]. Therefore, many restrictions are imposed on pesticide application in many countries around the world [9,12].

Nowadays, consumers concerned about fungicide residues in small fruits demand safer alternatives to replace synthetic pesticides [13]. Therefore, new disease control technologies that are safe for humans and the environment are needed [12]. Biological control, such as the use of plant volatiles is an exciting alternative [8]. Plants synthesize an enormous number of volatiles (phenols or their by-products). Many of them are responsible for flavor, while some have useful medicinal compounds [12].

Natural plant products tend to have low mammalian toxicity, broad-spectrum antimicrobial activity, are less hazardous than synthetic compounds, and are generally more acceptable to the public [4,7,12,14,15,16]. At present, there are many research results on the efficacy of plant volatiles to control fungal and microbial growth in in vitro (laboratory) conditions [17,18], but very few discuss their efficacy to extend the shelf life of fresh produce.

In this review, we discuss the efficacy of volatiles (single compounds) and essential oils (EOs, group of compounds) produced by plants to control food-borne bacteria (*Salmonella* spp., *Escherichia coli,* and *Listeria*) and fruit pathogens (mainly *Botrytis* and *Colletotrichum*) on extending the shelf life of small fruits, such as strawberries (*Fragaria* × *ananassa* Duch), raspberries (*Rubus* spp.), blueberries (*Vaccinium* spp.) and grapes (*Vitis vinifera* L.). We also provide insight into their in vitro, in vivo (in storage) and In situ (in the field) applications, and their mechanisms of action. A summary of the papers reviewed is presented in Table 1.

## 2. Control of Food Born Bacteria by Volatiles and Essential Oils in Small Fruits

Most of the studies on the application of natural volatiles and essential oils (EOs) to control foodborne pathogens, such as *Salmonella Typhimurium, Salmonella enterica*, *E. coli*, and *Listeria monocytogenes*, etc., have been conducted in vitro in petri dishes [31]. However, very few reports discuss the efficacy of plant volatiles and essential oils to control foodborne bacteria on small fruits during storage. Sun et al. [30] studied chitosan coating mixed with six different essential oils in vitro to control *E. coli* and *Penicillium digitatum* in blueberries during storage. Carvacrol and cinnamaldehyde had high antimicrobial capacity and were selected for in vivo studies to control blueberry pathogens. 

Sánchez-González et al. [17] tested biodegradable coatings with and without bergamot essential oil (from *Citrus × bergamia* Risso & Poit.) on table grapes (*V. vinifera* L.) during storage. They found that incorporation of bergamot essential oil improved the antimicrobial activity of the coatings and significantly reduced mold, yeast, and mesophile counts.

## 3. Control of Fungal Diseases in In Vitro Conditions

### 3.1. Effect of Volatiles on Fungal Diseases in In Vitro

Plant volatiles are mainly aldehydes, alcohols, acids and esters synthesized through the hydroperoxide lyase (HPL) and liposygenase (LOX) pathways. These products are involved in plant wounding responses and may have physiological roles (such as self-defense and antimicrobial properties) beside their role in aroma biosynthesis [9].

The effect of single compound terpene, terpenoids, and aldehyde vapors and their mixture from crushed tomato (*Solanum* sp. ‘Mountain Pride’) leaves were tested on conidiospores from *Alternaria alternata* and *Botrytis cinerea*. (E)-hex-2-enal and hexanal inhibited *A. alternata* hyphal growth and *B. cinerea* spore growth, while the terpene limonene and 2-carene did not affect hyphal growth [19]. However, the synergistic effect of other bioactive volatiles should not be excluded. The unsaturated aldehydes (E)-non-2-enal and (E)-hex-2-enal, were significantly more active than saturated hexanal, in inhibiting fungal growth [19].

Similarly, Arroyo et al. [9] examined the antifungal activity of eight aroma compounds of strawberry fruit against *Colletotrichum acutatum in vitro*. The aldehyde (E)-hex-2-enal significantly inhibited mycelial growth and spore germination (minimum inhibitory dose, MID of 33. 6 μL L^−1^). Hexanal was only effective when applied in higher doses of up to 10 times [9]. The inhibitory concentrations of hexanal on pathogens reported by others are quite different. Almenar et al. [14] observed that hexanal completely inhibited the growth of *C. acutatum*, *B. cinerea*, and *A. alternata* at low concentrations of 1.1, 1.3 and 2.3 μL L^−1^ air respectively. Andersen et al. [32] reported that 0.00007 μL L^−1^ air of hexanal reduced *A. alternata* growth on agar by 50%. Song et al. [33] reported that 0.5 μL L^−1^ air inhibited *B. cinerea* growth on potato dextrose agar (PDA ) media and Archbold et al. [24] showed 0.008 μL L^−1^ (2 μL/250 ml container) hexanal reducing *B. cinerea* in inoculated strawberries by 90%. In contrast to all these reports, Hamilton-Kemp et al. [19] observed a stimulatory effect of hexanal on *A. alternata* and *B. cinerea* mycelial growth at low levels.

In an experiment, among five antifungal volatiles (hexan-1-ol, benzaldehyde, 2-nonanone, (Z)-hex-3-en-1-ol and (E)-hex-2-enal) released by raspberry and strawberry fruits, (E)-hex-2-enal and benzaldehyde were the most effective against three fungal (*A. alternata*, *B. cinerea*, and *Colletotrichum gloeosporioides*) species in vitro [20].

To our knowledge, only one study reported the application of slow-release hexanal in postharvest fungal control in small fruits. To develop a controlled release mechanism, hexanal was incorporated into a polymer (β-cyclodextrin) and then evaluated in vitro against *B. cinerea*, *C. acutatum* and *A. alternata*. Hexanal was fungistatic on all three pathogens and fungicidal on *C. acutatum*. *C. acutatum* was more responsive to hexanal than the other two pathogens. The data suggested that a slow-release compound provided a more uniform volatile dose during storage and was more effective in reducing postharvest diseases [14].

### 3.2. Effect of Essential Oils on Fungal Diseases In Vitro

Essential oils (EO) are naturally occurring substances extracted from plant material and are a complex of volatiles produced in different plant parts. They constitute different plant secondary metabolites mainly from terpenes, and other aromatic compounds [34]. They have various functions in plants such as resistance against pest and diseases [13]. Essential oils with active ingredients can be used as an alternative management method to control microorganisms. They have shown antibacterial, antifungal, and antioxidant properties against some important food-borne and plant pathogens.

Essential oils have three main features. Since they are produced by plants, they are safer to the environment and humans than synthetic alternatives. Second, they have low risk of resistance development by pathogens because they are a mixture of oil components and pathogens lack the mechanism to develop resistance against a range of chemicals. Third, there is a broad range of natural compounds that have the potential to be used for controlling pathogens [7]. Essential oils are generally considered safe for treating food products, and sometimes even beneficial in extending their shelf life. However, phytotoxicity, off-flavors and off-odors have also been reported.

A large number of papers have been published on the biological activity of essential oils. The large variability among the data may be attributed to factors such as genetics, climate, geographical and seasonal conditions, distillation techniques and time of harvest [4]. Consequently, the chemical composition of essential oils needs to be standardized and reproducible [7].

Most of the reports on the inhibition of post-harvest fungal pathogens by essential oils focus on in vitro conditions [28]. The efficacy of several essential oils and their antimicrobial compounds were studied in extending the shelf life and inhibiting decay of strawberries. Only a limited number of literature investigated the effect of essential oils and/or plant extracts against *B. cinerea* or other small fruit pathogens [4,6,7,18].

Two studies [4,18] screened a large number of essential oils and plant extracts for their efficacy against *B. cinerea*. Wilson et al. [18] screened 345 plant extracts for their antifungal activity and thirteen were highly antifungal. The extracts from *Capsicum* and *Allium* genera were the most effective and inhibited *B. cinerea* spore germination after 48 and 24 hours, respectively. Among 49 essential oils tested, red thyme (*Thymus zygis* L.), palmarosa (*Cymbopogon martini* (Roxb.) Will. Watson), clove buds (*Syzygium aromaticum (L.) Merr. & L.M. Perry*), and cinnamon leaf (*Cinnamomum zeylanicum* Blume) greatly inhibited germination of *B. cinerea* spores at the lowest concentration tested (0.78% dilution; initial concentration was not provided). The essential oils with high antifungal activity and the highest frequency were: cineole, limonene, α-pinene, β-myrcene, camphor and β-pinene [18].

Tripathi et al. [4] tested essential oils of 26 plants against B. cinerea. Among them, ten plants (*Zingiber officinale* Roscoe, *Z. cassumunar* Roxb., *Prunus persica* (L.) Batsch, *Ocimum sanctum* L., *Ocimum gratissimum* L., *Ocimum canum* Sims, *Lawsonia inermis* L., *Eupatorium cannabinum* L., *Eucalyptus citriodora* Hook., and *Chenopodium ambrosioides* L.) showed 100% toxicity at 500 (mg L^−1^). The essential oils were a mixture of nine major and sixteen minor compounds with possible synergistic effects between major and minor constituents [4].

In another study, growth of *B. cinerea* mycelium was completely inhibited by thyme, oregano, dictamnus and marjoram essential oils at 150, 200, 200 and 300 mgml^-1^, respectively. The main component of oregano oil was thymol, and the main component of marjoram, thyme and dictamnus oils was carvacrol [7].

Bhaskara Reddy et al. [6] reported that, there is a significant difference between the antifungal activity of two clones of *Thymus vulgaris* (Laval-1 and Laval-2) against two strawberry storage pathogens (*Rhizopus stolonifer* and *B. cinerea*). Oil from Laval-2 clones showed higher antifungal activity in vitro and decay control action in vivo which is mainly due to its higher carvacrol, thymol and linalool contents. Lemongrass oil reduced germ tube length and spore germination in *R. stolonifer*, *Colletotrichum coccodes*, and *B. cinerea* [13].

Combrinck et al. [21] tested eighteen essential oils on five pathogens, including *B. cinerea* isolated from grapes. *B. cinerea* was the most susceptible to cinnamon, thyme, and eugenol at the lowest concentration tested (500 μL L^−1^).

In the study conducted by Duduk et al. [2], clove bud, thyme and cinnamon bark essential oils were fungistatic on mycelial growth of *C. acutatum* (667 μL L^−1^ of medium) and their volatiles disabled appressoria formation (1.53 μL L^−1^ of air) and prevented conidia germination (76.5, 15.3 and 1.5 μL L^−1^ of air respectively).

Santos et al. [22] tested the efficacy of chitosan packages incorporated with *Origanum vulgare* L. essential oil on *Aspergillus niger* and *R. stolonifer* on grapes. Mycelial growth was inhibited by essential oil in in vitro conditions.

Bouchra et al. [23] evaluated essential oils of seven Labiatae plants grown in Morocco for their antifungal activity in in vitro conditions against *B. cinerea*. *Thymus glandulosus* Req. and *Origanum compactum* Benth., with thymol and carvacrol as main compounds, inhibited mycelium growth more effectively than others.

### 3.3. Mechanism of Action of Volatiles and Essential Oils on Pathogen Growth in In Vitro Conditions

Different concentrations of hexanal are effective against fungal growth. Caccioni et al. [35] believed that, the actual vapor pressure rather than the concentration of volatiles determines their effectiveness. Therefore, the effectiveness of hexanal depends on its vapor pressure (effective concentration), its initial amount and the tested fungus. In essential oils, the antifungal activity strongly depends on the proportion of the individual components [36].

Gardini et al. [37] posited that hexanal is not soluble in water and therefore in the culture medium. However, Almenar et al. [14] showed that PDA medium absorbed hexanal and prevented *A. alternata* and *C. acutatum* growth (3.0 and 3.3 μL respectively). Therefore, if media can absorb henxanal, we hypothesize that fresh produce can also absorb small amounts of volatiles and extend their shelf life by extruding it. 

The saturation status of volatile compounds seems to also affect their efficacy with unsaturated compounds being more reactive than saturated ones. Hamilton-Kemp et al. [19] and Andersen et al. [32] demonstrated that (E)-hex-2-enal (unsaturated aldehyde) was more effective against *B. cinerea* and *A. alternata* hyphal growth than hexanal (saturated aldehyde). Arroyo et al. [9] showed that aldehydes ((E)-hex-2-enal and hexanal) which are less saturated than alcohols and esters, are more effective in fungal inhibition. In contrast, among the alcohols tested, (E)-hex-2-en-1-ol, and (Z)-hex-3-en-1-ol which are less saturated than hexan-1-ol, showed lower antifungal activity against *C. acutatum* mycelial growth [9]. The reason could be due to a different reaction of *C. acutatum* to volatiles compared to *B. cinerea* and *A. alternata* as was mentioned by Almenar et al. [14]. Arroyo et al. [9] studied the conidial cells of *C. acutatum* treated with (E)-hex-2-enal by transmission electron microscopy. They observed that cell components were highly disorganized and cell wall and plasma membrane were disrupted, resulting in lysis of organelles and cell death. 

Almenar et al. [14] reported that *B. cinerea* and *A. alternata* reacted to hexanal similarly but different from *C. acutatum*. They suggested that the inhibition mechanisms are different, but no further explanation was provided. However, Hamilton-Kemp et al. [19] reported that *C. acutatum* and *B. cinerea* responded similarly meaning hyphal growth was inhibited by aldehyde but not terpenes. However, they suggested that more cautious interpretation is needed as variable culture conditions and organism strain may affect the results.

The difference in the affinity of these compounds with microbial membranes seems to affect their effectiveness and their toxicity [14]. Cell membrane permeability and its interaction with volatiles are key elements in the effectiveness of gaseous compounds [38]. Changes in the permeability of cell membranes, and degeneration of the ion gradients adversely affect vital cell processes and lead to cell death [16].

In this regard, Kulakiotu et al. [8] reported cell protoplast secretion without initial cell wall disruption in mycelial hyphae of *B. cinerea* exposed to volatiles of grape (*Vitis labrusca,* cv. Isabella) resulting in cell wall deformation. Hyphae from the control treatment was healthy, possessing conidiophores with abundant conidia [8]. The mechanism of action of volatiles inhibiting hyphal growth and their effect on membranes needs further investigation. Thymol and carvacrol also showed antimicrobial activity against natural grape yeasts in red wines via damage to the membrane, leakage of cytoplasmic content and finally inhibition of erogosterol biosynthesis [39].

The fact that different natural compounds have a different antifungal profile suggests that different modes of action exist. In human pathogens, several mechanisms of actions have been reported for antifungal activity of natural plant compounds. Sivakumar et al. [40] reported that chalcones (aromatic ketons found in a few plant species) are able to disrupt fungal cell wall formation and cell lysis by inhibiting synthesis of the cell wall polysaccharide 1,3-beta-D-glucan. They can also interrupt cell division by inhibiting the conversion of tubulin into microtubules [41].

Aldehydes, another group of volatiles, inhibit fungal cell division by reacting with, and inactivating sulfhydryl, the functional group involved in fungal cell division. Some aldehydes (such as cinnamaldehyde, citral and perillaldehyde) are good electron acceptors and form a charge transfer complex with electron donors present in fungal cells, thus interfering with fungal metabolism [42].

Volatiles with α,β-unsaturated cabonyl groups (such as enones and enals) often react with nucleophilus in fungi through the Michael reaction to create chemical modifications and interrupt fungal growth [43] of *C. acutatum, Colletotrichum fragariae, C. gloeosporioides, Fusarium oxysporum, B. cinerea*, and *Phomopsis* sp. Nevertheless, the accessibility of α,β-unsaturated carbonyl groups to bulky nucleophile biomolecules plays an important role in their antifungal activity [44].

It has been reported that mitochondrial electron-transfer respiration systems in mammals were inhibited by *C. ambrosioides* essential oil component such as carvacrol [36]. Another component of the EO (ascaridole) formed toxic radicals in the presence of Fe^2+^ and reduced hemin [36]. The phenol carvacrol has shown antibacterial properties due to its ability to distribute into membranes and to permeate in them causing a breakdown of ion gradients. Carvacrol also increases the passive permeability of the cell membrane and modulates certain Ca^2+^ permeable transient receptor potential channels, inhibits sarcoplasmic reticulum Ca^2+^ ATPase, and activates ryanodine receptors in skeletal muscle, thereby influencing intracellular calcium homeostasis (Monzote et al. [36] and references therein).

## 4. Control of Fungal Diseases in In Vivo (in Storage) Conditions

### 4.1. Effect of Volatiles on Fungal Diseases In Vivo

Arroyo et al. [9] reported complete inhibition of *C. acutatum* development after five days by (E)-hex-2-enal in inoculated strawberry fruit. This compound also effectively reduced *B. cinerea* disease symptoms in grapes, blackberry (*Rubus* spp.) and strawberry [24]. Vaughn et al. [20], evaluated fifteen volatiles from strawberries and red raspberries during ripening and found that five compounds (hexan-1-ol, benzaldehyde, 2-nonanone, (Z)-hex-3-en-1-ol and (E)-hex-2-enal) completely inhibited *A. alternata*, *B. cinerea*, and *C. gloeosporioides* on strawberry and raspberry fruits.

In another study, treatments with thymol, eugenol, and menthol reduced decay in strawberries, with thymol being the most effective at slowing berry decay compared to the other two compounds [16]. Similarly, p-cymene, linalool, carvacrol, anethole, and perillaldehyde effectively retarded blueberry mold formation, while cinnamic acid and cinnamaldehyde were less effective in suppressing blueberry mold growth [25]. It has also been reported that grape (cv. Isabella) volatiles reduce *B. cinerea* activity and inoculum density, thereby limiting incidence and infection in kiwifruits [8].

### 4.2. Effect of Essential Oils on Fungal Diseases In Vivo

Essential oils from a number of plants have shown activity against strawberry pathogens. For example, eucalyptus (*Eucalyptus globulus* L.) and cinnamon oil (*C. zeylanicum*, Blume) improved fruit quality and reduced fruit decay [12], while cinnamon bark EO reduced both *C. acutatum* and *B. cinerea* penetration, development and number of infected fruits at concentrations above 76.5 μL L^−1^ of air [2]. Thyme (*T. vulgaris* L.) EO had higher antifungal activity and suppressed pathogen development at concentrations above 15.3 μL L^−1^ of air. At a higher concentration (153 μL L^−1^ of air), thyme EO inhibited *C. acutatum* development on inoculated fruits [2]. Also, strawberry decay caused by *R. stolonifer* and *B. cinerea* was controlled by volatiles of *T. vulgaris* [6,28] with both reports showing differences between thyme genotypes in terms of chemistry and efficacy. 

Essential oils have also been tested on grape diseases. In a laboratory study, Walter et al. [26] found that EOs from clove (*S. aromaticum (L.) Merr. & L.M. Perry*), thyme, and massoialactone (derived from the bark of the tree *Cryptocarya massoia* R.Br.) significantly reduced necrotic lesions on grape leaves caused by *B. cinerea* when treated with thyme, and massoialactone. In a separate study, Valero et al. [27] showed that thymol or eugenol (volatile from clove) reduced the loss of sensory quality and microbial spoilage of grape in modified atmosphere storage. 

Chitosan packages infused with *O. vulgare* essential oil changed spore and mycelia morphology and inhibited growth of *A. niger,* and *R. stolonifer* spore germination on inoculated grapes in storage [22]. The oils of *Z. officinale*, *O. sanctum* and *P. persica* were also effective in controlling storage rot of grapes during in vivo trials, with treated grapes showing an improved shelf life for up to 6 days [4].

### 4.3. Effect of Volatiles and Essential Oils on Fruit Quality

Wang et al. [16] found that treatment with thymol and eugenol extended strawberry shelf life and increased fruit free radical scavenging capacity, thereby enhancing resistance to spoilage and deterioration. Essential oil treated fruits were found to have higher amounts of sugars, flavonoids, anthocyanins, organic acid and phenolic compounds. The increase in phenolic content may have led to increased oxygen radical absorbance capacity (ORAC) and decreased fruit spoilage in essential oil treated strawberries [16]. 

Carvacrol, anethole, and perillaldehyde also enhanced antioxidant activity and increased total anthocyanins, phenolics and hydroxyl radical (•OH) scavenging capacity in blueberry fruit tissues [25]. Chitosan coating with carvacrol and cinnamaldehyde was also found to maintain blueberry firmness and effectively extended blueberry shelf life [30].

Similarly, postharvest essential oil treatments (perillaldehyde, linalool, cinnamaldehyde, cinnamic acid, anethole, and carvacrol) enhanced antioxidant capacity in raspberries with perillaldehyde being the most effective [29]. 

Grape berries in a modified atmosphere package with eugenol or thymol had lower weight loss, lower changes in skin color, reduced ripening, and lower decay throughout storage [27]. Lower weight loss was also seen in gerbera (*Gerbera jamesonii* cv. Dune) flowers treated with thymol [45].

Sánchez-González et al. [17] tested chitosan-coated packages of grapes alone or in mixture with bergamot essential oil and found chitosan mixed with bergamot oil to be the most effective to control respiration rate, water loss and antimicrobial activity during storage. Chitosan packages infused with *O. vulgare* essential oil also preserved the quality, physical, physiochemical and sensory attributes of grapes in storage [22].

### 4.4. Mechanism of Fruit Resistance to Fungal Attack Treated by Volatiles and Essential Oils 

Plant volatiles play a major role in self-defense. Pe´rez et al. [46] observed that a 25% decrease in (E)-hex-2-enal content during strawberry development and ripening coincides with activation of latent infection, and appearance of visible disease symptoms, leading to extensive fruit damage.

Six and nine-carbon volatiles are produced in plant tissues in response to wounding in LOX (liposygenase) and HPL (hydroperoxide lyase) pathways. In addition to their role in aroma biosynthesis, products of LOX and HPL pathways, have antimicrobial and antifungal activities and may have a role in plant-pathogen interactions. Some have also shown a stimulatory effect on some pathogenic fungi. In *Arabidopsis thaliana*, (E)-hex-2-enal activated several self-defense genes (i.e., lipoxygenase 2 and allene oxide synthase), lignified leaves and accumulated pathogenesis-related proteins (i.e., 3 transcripts, and camalexin), which reveals that volatile treatments stimulate a wound-repair mechanism, and will ultimately act as a physical barrier to the pathogen penetration [47]. Externally applied essential oils improved fruit resistance to fungal attack, mostly due to increased antiproliferative activity and free radical scavenging capacity in strawberries [16]. This has also been reported in kiwifruit (*Actinidia deliciosa* cv. ‘Hayward’) against *B. cinerea* with the application of grape (cv. Isabella) volatiles which induced mechanisms of resistance in the host [8].

Since plant volatiles inhibit fungal growth and are produced rapidly through the lipoxygenase pathway (in response to wounding), more studies are needed to further clarify their role in plant self-defense systems.

In addition to self-defense and induced resistance, essential oils affect pathogens by contact either in media or as volatiles in storage. Essential oils also lead to the secretion of cell protoplasts without previous cell wall disruption, as shown by the presence of chlamydospores and deformation of cell walls in *B. cinerea* hyphae treated with grape (cv. Isabella) volatiles [8]. 

The bioactivity of essential oils in the vapor phase is a very attractive characteristic that makes them suitable for use as fumigants in small fruits not suited to aqueous sanitation [16]. Easy application of essential oil vapors during storage makes them more attractive than dipping [48]. Essential oil application could also limit the spread of pathogens by suppressing spore production or reducing spore load on surfaces or in storage. However, the role of volatiles in spore germination and pathogen infectivity needs to be understood in order to develop new techniques for disease control [8].

### 4.5. Phytotoxicity, Off-Flavor and Off-Odor of Volatiles and Essential Oils on Fresh Produce

Extended exposure to volatiles has resulted in phytotoxicity on strawberry fruits and affected fruit quality [9]. The phytotoxicity of (E)-hex-2-enal has also been described on pears [49], beans [50], and sliced apples [51]. Volatiles 2-nonanone, hexan-1-ol and benzaldehyde slightly damaged strawberry fruit while (Z)-hex-3-en-1-ol and (E)-hex-2-enal caused extensive tissue necrosis [20]. Fallik et al. [52] reported strawberry weight loss and other side effects by (E)-hex-2-enal. Walter et al. [26] reported excessive water loss from detached grape branches (in the laboratory, but not in the field) exposed to thyme and massoialactone, and phytotoxicity on grape flowers in the field. Also, incorporation of bergamot essential oil in chitosan coating resulted in brown-colored grapes during storage [17].

In the authors’ preliminary experiments with the application of thymol and carvacrol in air-tight containers, phytotoxicity was observed as increased mushiness and dullness of strawberry fruits after 24 hours (unpublished data). However, Bhaskara Reddy et al. [6] did not observe any visual phytotoxic symptoms after 4 days of fruit exposure to *T. vulgaris* essential oils. Further work is needed to identify and minimize the cause of adverse effects on fruit quality by volatiles [9].

Very few studies have reported on the sensory characteristics of fruits treated with essential oils. Neri et al. [47] reported off-odors in peach and nectarine fruits treated with (E)-hex-2-enal described as a “green” (leafy) and “butyric” aroma. However, off-odors decreased during ripening and nothing was perceived after ripening. No fruit off-odors were observed in peach and nectarine fruits treated with carvacrol or citral. Aloui et al. [53] reported complete absence of off-flavors and off-odors in dates treated with citrus essential oils. Serrano et al. [54] treated cherries with eugenol, thymol or menthol and reported no sensory effects. However, cherries treated with eucalyptol generated off-flavors. Prasad & Stadelbacher [55], reported that acetaldehyde treated strawberries did not show any off-flavor at a concentration of 1%, but showed off-flavor at 4%.

The reviewed articles suggest that most of the essential oils do not leave off-flavors or off-odors on intact fruits at the minimum inhibitory concentrations, especially when stored fruits finish their ripening process. However, there is variation between fruit species and cultivars and each commodity has to be tested individually. For example, Neri et al. [47], reported that stone fruits were more sensitive to (E)-hex-2-enal injury than pome fruits and showed off-flavor, probably because they absorb more (E)-hex-2-enal than pome fruits.

## 5. Control of Fungal Diseases in the Field

To our knowledge, only one research paper described the application of essential oils in the field. A single application of thyme oil or massoialactone application on grape bunches and leaves reduced *B. cinerea* in the field trial [26]. However, the efficacy of Thyme oil to control bunch rot needs further field evaluations, as floral tissue browning occurred [26].

## 6. Conclusions

In this review, the efficacy of essential oils (plant-based multi-compounds) and single compound volatiles was discussed in in vitro, in vivo (in storage) and In situ (in the field) conditions. Despite the importance of fresh fruits food safety, there are not many research articles on the effectiveness of essential oils to control bacteria populations on small fruits, which suggests the need for research in this area. Many research articles discussed the positive effects of volatiles and essential oils in in vitro conditions on media-grown fungal populations with a large variation in their efficacy and their minimum inhibitory concentrations. Most of these variations were related to the vapor pressure of volatiles. 

The efficacy of multi-compound essential oils is even more variable due to genetic variation, environmental conditions, and synergistic effects of multiple compounds. Major compounds play an essential role in the antimicrobial activity of essential oils, however, minor compounds in the mixture may have synergistic effects. Future studies are needed to explain the role of single natural compounds or their combination in plant-fungal interactions.

(E)-hex-2-enal and hexanal were the most effective volatiles and thyme the most effective essential oil in controlling fungal diseases of small fruits both in vitro and in storage. Thyme oil has the potential as a postharvest treatment to extend shelf life of fresh fruits and vegetable and to replace commercial fungicides or controlled atmosphere storage. However, active packaging and improved formulation techniques are needed to prolong activity, reduce volatility and improve coverage.

Several researchers have mentioned the toxicity of volatiles and essential oils after extended exposure. Research is needed to study the effect of volatiles and essential oils on vegetative and reproductive phases of fungal development, fruit quality (firmness, sensory evaluations), cell wall structure and integrity and their role in plant self-defense and postharvest storage.

## Figures and Tables

**Table 1:** in vitro, in vivo (in storage), and In situ (in field) applications of volatiles (single compounds) and essential oils (EOs, multiple compounds) on diseases and plants, and parameters measured. The abbreviations are explained in the notes.

Volatile, EO, plant extract	Concentration	Disease/plant	Parameters measured	Reference
**in vitro application**				
(E)-hex-2-enal, hexanal, (E)-non-2-enal, nonanal, 2-carene, limonene and aldehydes upon wounding of tomato leaves	10% solution (*w*/*w*) of the test compound and diluted serially (*w*/*w*) to 1 and 0.1% solutions.	*Altenaria alternata* and *Botrytis cinerea*	Hyphal length	Hamilton-Kemp et al. [19]
Aldehydes hexanal and (E)-hex-2-enal; the alcohols hexan-1-ol, (Z)-hex-3-en-1-ol, and (E)-hex-2-en-1-ol; and the esters (Z)-hex-3-enyl acetate, (Z)-hex-2-enyl acetate, and hexyl acetate.	33.78 to 1351.35 µL L^−^^1^	*Colletotrichum acutatum*	Mycelial growth, conidial germination, development of appressoria, MID, ID95, ID50	Arroyo et al. [9]
Fifteen compounds from aldehydes, alcohols, ketones, an ester and a mixed alcohol and ketone moiety.	0, 0.02, 0.04, 0.1, 0.4 µL mL^−1^	*B. cinerea*	Growth of fungi, % of control	Vaughn et al. [20]
Hexanal in β-cyclodextrin complex	0, 1, 1.5, 2, 4, 5, 7, 10 µL	*C. acutatum*, *A. alternata* and *B. cinerea*	Radial growth of cultures in cm^2^	Almenar et al. [14]
Extracts from 345 plants and 49 essential oils	10% plant extract solution, 50, 25, 12.5, 6.25, 3.13, 1.56, 0.78, and 0.39% EOs	*B. cinerea*	Reduction in spore germination	Wilson et al. [18]
Twenty six essential oils of ten plants (*Chenopodium ambrosioides*, *Eucalyptus citriodora*, *Eupatorium cannabinum*, *Lawsonia inermis*, *Ocimum canum*, *O. gratissimum*, *O. sanctum*, *Prunus persica*, *Zingiber cassumunar* and *Zingiber officinale*)	500 ppm, different for MIC and MFC	*B. cinerea*, and only three selected essential oils on 15 other fruit rotting pathogens	% mycelial inhibition, MIC	Tripathi et al. [4]
Oregano, thyme, dictamnus, marjoram (carvacrol), lavender (linalool, linalyl acetate), rosemary, sage (eucalyptol) and pennyroyal (cis-menthone) EOs	Not specified	*B. cinerea*, *Fusarium* sp., *Clavibacter michiganensis*	Radial growth on PDA	Daferera et al. [7]
EOs of two clonal types of *Thymus vulgaris*	50, 100, 200 ppm	*B. cinerea* and *Rhizopus stolonifer*	% inhibition of radial growth	Bhaskara Redy et al. [6]
Lemongrass (*Cympopogon citratus*)	25, 50, 100, 500 ppm	*B. cinerea*, *Colletotrichum coccodes*, *C. herbarum*, *R. stolonifer*, *Aspergillus niger*	Pathogen development, spore production, spore germination, germ tube length	Tzortzakis & Economakis [13]
Eighteen EOs	50–3000 µLL^−1^	5 pathogens from 5 crops including *B. cinerea* from grape	Visual inspection, inhibition of mycelial growth (%)	Combrinck et al. [21]
Thyme (P-cymene, thymol, α-terpineol, carvacrol, Cinnamon bark (cinnameldehyde, cinnamyl acetate), Clove bud (eugenol, β-caryophyllene)	13 concentrations from 0.067 to 667 µL L^−1^ of media	*C. acutatum*	Mycelial growth, conidial germination, appressoria formation	Duduk et al. [2]
*Origanum vulgare* L. essential oil	40, 20, 10, 5, 2.5, 1.25, 0.06 µL mL^−1^	*R. stolonifer* and *A. niger*	Mycelial growth of the test fungi, spore germination and morphological changes	Santos et al. [22]
Essential oils of seven Moroccan Labiatae	0, 10, 50, 100, 150, 200 and 250 ppm	*B. cinerea*	Percentage of inhibition of radial growth vs control	Bouchra et al. [23]
**in vivo (in storage) application**				
(E)-hex-2-enal	4, 5, 10, 20, and 50 µL L^−1^	*C. acutatum* inoculated strawberry fruit	Incidence of infected fruits, scale 0,1	Arroyo et al. [9]
Three groups of naturally occurring volatile compoundscontains 24 volatiles	2, 10, 100 µL/250 mL bottle	Strawberry, blackberry & grape	Lesion appearance and size, phytotoxicity	Archbold et al. [24]
Fifteen volatiles released by red raspberries and strawberries	0.4 µL mL^−1^	*B. cinerea* on raspberry and strawberry	Rated for development of fungi and damage of volatile	Vaughn et al. [20]
Thymol, menthol, eugenol	200 mg L^−1^	Strawberry	Sugar, acid, anthocyanin, TPC, ORAC, DPPH, HRS, SARS, flavonoids	Wang et al. [16]
Carvacrol, anethole, cinnamaldehyde, cinnamic acid, perillaldehyde, linalool, and p-cymene	200 mg L^−1^	Blueberries	ORAC) and hydroxyl radical (•OH) scavenging, total anthocyanins, total phenolics capacity, sugars, organic acids, % of fruit showing fungal symptoms	Wang et al. [25]
Volatile substances emitted by ‘Isabella’ grapes	0, 300, 400, or 500 g of ‘Isabella’ grapes	*B. cinerea* in kiwifruit	# infected kiwifruit, # kiwifruit on which fungal fruiting bodies had appeared	Kulakiotu, et al. [8]
Eucalyptus and cinnamon EOs	50, 500 ppm	Strawberry, tomato	Degree of visual infection, weight loss, TSS, firmness, TA, TPC,	Tzortzakis [12]
Thyme, Cinnamon bark, Clove bud EO	13 conc. (0.067–667)	*C. acutatum*	# of diseased fruit or mycelial growth	Duduk et al. [2]
Essential oils from thyme (*T. vulgaris*), clove (*Syzygium aromaticum*), and massoialactone (bark of the tree *Cryptocarya massoia*)	(0, 0.033, 0.1, 0.33, 1.0 and 3.3%) for phytotoxicity, *B. cinerea* inoculated on heated lesions and treated with (0.033, 0.1, 0.33%) EOs	*B. cinerea* in grapes	Scaling the formation of necrosis on the underside of the leaves	Walter et al. [26]
Eugenol or thymol	75 or 150 µL/bag (vol. was not mentioned)	Grape	Ethylene, weight loss, color and firmness, TSS, TA, sensory analysis, decay, microorganism analysis, antioxidant activity, TPC, total anthocyanins, organic acids, and sugar contents	Valero et al. [27]
*O. vulgare* L. essential oil	40, 20, 10, 5, 2.5, 1.25, 0.06 µL mL^−1^	Grapes	TSS, TA, weight loss, color, firmness, anthocyanin, and sensory characteristics of the fruits during storage	Santos et al. [22]
*O. sanctum*, *P. persica* and *Z. officinale*	200,100 and 100 ppm	*B. cinerea* in grapes	Initiations of rotting of the fruits	Tripathi et al. [4]
*Thymus danensis* and *T. carmanicus*	150, 300, 600, 1200 µL L^−1^	*R. stolonifer*, *Penicillium digitatum*, *A. niger* and *B. cinerea* strawberry	Disease incidence (%)	Nabigol & Morshedi [28]
EOs of two *T. vulgaris* clones	50, 100, 200 ppm	*B. cinerea* and *R. stolonifer*	Decay of fruit	Bhaskara Reddy et al. [6]
Carvacrol, anethole, cinnamic acid, perillaldehyde, cinnamaldehyde, and linalool	200 mg L^−1^	Raspberries	SOD, CAT, G-POD, AsA-POD, GR, GSH-POD, MDAR, DHAR, Protein content, TPC, Total anthocyanins, ORAC, HOSC, DPPH, flavonoids	Jin et al. [29]
Carvacrol, cinnamaldehyde	0.50%	*Escherichia coli* and *P. digitatum* on blueberries	Microbial populations, fruit firmness	Sun et al. [30]
Bergamot EO, on grape	2% *w*/*v*	Grape cv Muscatel	Microbial counts, weight loss, ºBrix, total phenols, antioxidant activity, color and texture, respiration rate	Sánchez-González et al. [17]
**In situ (field application)**				
Thyme R oil in two years and massoialactone year 2	0.033 Thyme, 0.1 massoialactone	*Botrytis* in grape	*B. cinerea* sporulation on leaves, % of berries showing *B. cinerea* sporulation	Walter et al. [26]

Notes: The aforementioned abbreviations stand for PDA (Potato dextrose agar), MIC (Minimum inhibitory concentration), MID (minimum inhibitory doses), ID (inhibitory doses), ORAC (Oxygen radical absorbance capacity), TPC(Total phenolic content), HRS (Hydroxyl Radical Scavenging), SARS (Superoxide Anion Radical Scavenging), TSS (Total soluble solids), TA (Titratable acidity), SOD (Superoxide dismutas, EC 1.15.1.1), CAT (Catalase, EC 1.11.1.6), G-POD (Guaiacol peroxidase, EC 1.11.1.7), AsA-POD (Ascorbate peroxidase, EC 1.11.1.11), GR (Glutathione reductase, EC1.6.4.2), GSH-POD (Glutathione peroxidase, EC 1.11.1.9), MDAR (Monodehydroascorbate reductase, EC 1.6.5.4), DHAR (Dehydroascorbate reductase, EC 1.8.5.1), HOSC (Hydroxyl radical scavenging capacity (ºOH; HOSC) assay), DPPH (2,2-Di (4-tert-octylphenyl) -1-picrylhydrazyl (DPPH) scavenging capacity assay).
